# Construction of Intelligent Recognition and Learning Education Platform of National Music Genre Under Deep Learning

**DOI:** 10.3389/fpsyg.2022.843427

**Published:** 2022-05-26

**Authors:** Zhongkui Xu

**Affiliations:** College of Music and Dance, Henan Normal University, Xinxiang, China

**Keywords:** deep learning, deep belief network, national music genre, Chinese traditional instrumental music, music feature extraction

## Abstract

In order to study the application of the deep learning (DL) method in music genre recognition, this study introduces the music feature extraction method and the deep belief network (DBN) in DL and proposes the parameter extraction feature and the recognition classification method of an ethnic music genre based on the DBN with five kinds of ethnic musical instruments as the experimental objects. A national musical instrument recognition and classification network structure based on the DBN is proposed. On this basis, a music library classification retrieval learning platform has been established and tested. The results show that, when the DBN only contains one hidden layer and the number of neural nodes in the hidden layer is 117, the basic convergence accuracy is approximately 98%. The first hidden layer has the greatest impact on the prediction results. When the input sample feature size is one-third of the number of nodes in the first hidden layer, the network performance is basically convergent. The DBN is the best way for softmax to identify and classify national musical instruments, and the accuracy rate is 99.2%. Therefore, the proposed DL algorithm performs better in identifying music genres.

## Introduction

At present, the classification and recognition system for music data search is mainly used to manually extract music features and then form classifier modeling. Finally, the established model is used to identify and classify music samples (Myna et al., 2020). However, there are some problems with manually extracting music features. It is very difficult to extract music features manually because different classification and recognition tasks require different music features. Sometimes, it is even impossible to name the required music features (Hernández-López et al., 2021). As a new feature extraction technology, deep learning (DL) has made great progress, such as in the fields of image processing and natural language understanding. Therefore, this exploration aimed to adopt more appropriate music features in national musical instruments’ music style recognition and classification methods. Music genre recognition (MGR) plays an important role in the context of music indexing and retrieval. Unlike images, music genres are composed of highly diversified direct features with different levels of abstraction. However, most of MGR’s representation learning methods focus on global features and make decisions from the same set of features. In order to make up for these defects, Ng et al. (2020) integrated the convolutional neural network with NetVLAD and self-attention to capture local information across levels and understand their long-term dependence. The meta classifier was used for the final MGR classification by learning from aggregated advanced features from different local feature coding networks. Experimental results show that this method has higher accuracy than other advanced models on ISMIR2004 and extended ballroom datasets.

According to interviews with two website administrators, Thorgersen (2020) surveyed the development of Whoa.nu learning platform for hip hop in Sweden. Hip hop education has now been institutionalized, just as jazz and rock once were institutionalized. It has changed from being rebellious and subversive to being accepted by the larger society and integrated into the academic community. The results here present the story of music learning in a subculture. Therefore, the proposed insights can help educators prepare for similar changes in the field of learning in the future music subculture. Ceylan et al. (2021) revealed that structural complexity described the time process of a specific value on different time scales. It was applied to audio characteristics to classify music files using the random forest and k-nearest neighbor (KNN) methods. Rahman et al. (2021) suggested that the classification of music genres with great attention in recent years was an important part of music information research because of the excellent deep neural network performance in computer vision. Some researchers used the Cable News Network (CNN) to classify music types. Although the above research has a certain research foundation in the field of music recognition, there are few research applications of DL in identifying music genres, and the recognition accuracy needs to be improved. Therefore, this exploration constructs a platform for an in-depth study of music recognition, which provides a new direction for the field of music recognition. Williamon and Antonini Philippe (2020) emphasized art, especially music, because performers in the two fields of sports and music must have a wide range of social and psychological needs, and the two have similarities. Harris and Küssner (2020) pointed out that social-emotional behavior was inextricably linked to our music experience. It reveals that there are many studies on music genre classification, but there are few studies on MGR using the DL method. This exploration can provide new research ideas for MGR.

The experimental analysis method is used to identify music genres. The research innovation is to propose a national MGR method based on a deep belief network (DBN), which shows the advantages of the DBN in absorbing features and improving the accuracy of music category recognition. The music genre learning platform built on this basis accurately reflects the performance advantages of the algorithm.

## Research Methods

### Extraction of Musical Features

The demand for searching, querying, and interactive access to the increasingly large online music database requires more reliable and faster tools to provide content analysis and description functions. For all information, genres are the key description of the music. They can be used to organize music collections in the form of categories in the music warehouse. Although the concept of genre is widely used, it is still an undefined concept, which makes automatic genre classification an important and challenging task. Music features can represent the basic attributes of musical redundancy, so it is essential to extract music features when identifying and classifying national musical instruments (Chen et al., 2018). The main classification feature of traditional national musical instruments in identifying national music genres is instrumental timbre, which mainly extracts short-term features (Lee et al., 2018). A frame refers to the short time in the voice signal, often only 10.30 ms per frame. During this period, the voice signal can be regarded as stable, and the music signal has similar characteristics. Therefore, the direct extraction of long-term features has a certain complexity, and it is usually realized by combining short-term features. Therefore, when people extract the original features of music signals, they mainly extract short-term features. There are three types of short-time features (Bansal et al., 2021). The music feature time domain is a kind of parameter feature, and it is directly extracted from the time-domain waveform of the music signal. It is widely used because of its intuitive processing and a small amount of calculation. The common characteristics of time-domain music are the short-time average cross zero ratio and short-term energy (Wang, 2020). The common characteristics of frequency-domain music are that the signals in the music field are converted from the time domain to the frequency domain by Fourier transform (Zhang et al., 2020). With the frequency domain processing and analysis of music signals, the obtained features are called the music feature frequency domain.

### Deep Belief Network for Deep Learning

**FIGURE 1:**
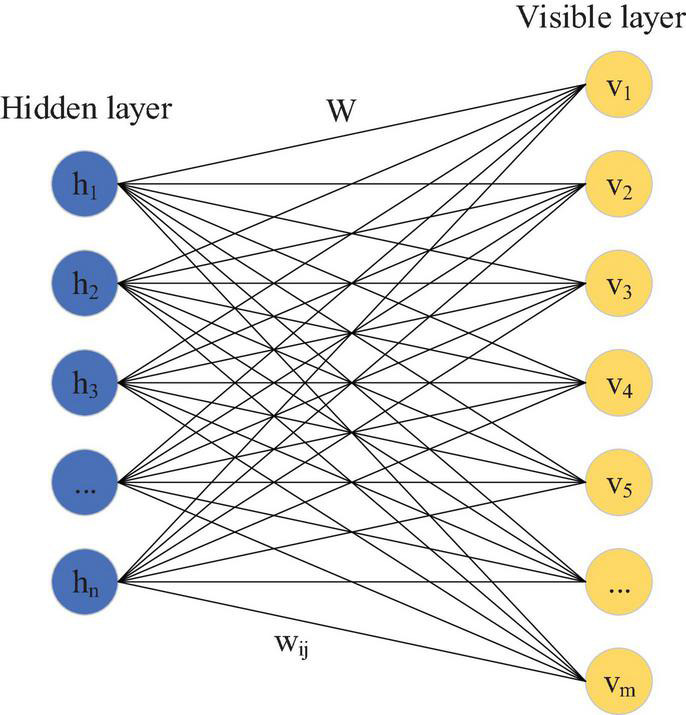
Structure of RBM.

The DBN is widely used as a DL algorithm (Patel et al., 2021). It is a generated probability model structure that is composed of many restricted Boltzmann machines (RBM) (Park and Yoo, 2020). Normally, it takes unidirectional data as input, and the audio data used to identify and classify national musical instruments are also unidirectional. Therefore, RBM is used as the DL algorithm. As an energy-based generative structure model, it is generally used to construct the DNN structure. This is a non-linear graph with two layers: one is the visible layer V and the other is the hidden layer h, in which the neuron nodes are fully connected between layers. As shown in Figure 1, there is no connection within the hidden layer, and there is no connection within the visible layer.

As Figure 1 shows, this RBM visible layer indicates that the number of input data nodes is m, and there are *n* hidden layer data nodes used to extract features. W represents the weight connection matrix of neurons between layers, and its function energy is calculated as follows:


(1) $$E\,\left(v,h,\theta \right)=-\sum_{i=1}^{m}\sum_{j=1}^{n}v_{i}\,h_{j}\,w_{\text{ij}}-\sum_{i=1}^{m}b_{i}\,v_{i}-\sum_{j=1}^{n}a_{j}\,h_{j}$$

*b_i_* represents the i-th visible layer offset, *v_i_* represents its unit, *a*_*j*_ represents the j–th hidden layer unit, *h*_*j*_ represents its offset, and *w*_ij_ represents the value connecting the j-th hidden layer and the i-th visible layer. The probability equation of the two layers is expressed as follows:


(2) $$p\,\left(v,h\right)=\frac{1}{Z}\,e^{-E\,\left(v,h\right)}$$

Its intermediate factor normalized *Z* can be expressed as:


(3) $$Z=\sum_{v,h}e^{-E\,\left(v,h\right)}$$

Therefore, the probability equation of the visible layer element is expressed as


(4) $$p\,\left(v\right)=\frac{1}{Z}\,\sum_{h}e^{-E\,\left(v,h\right)}$$

Because the RBM node is not connected in its own layer, all nodes connected to the hidden layer are only each visible layer node, and all points of the visible layer are only connected to each hidden layer node. Therefore, when determining the node state of one layer, the state nodes of another layer follow a separate conditional distribution.

### The Deep Belief Network-Based Ethnic Music Genre Recognition

The recognition and classification of music genres occupy a critical position in music information retrieval. Many music users are only interested in music of certain styles and genres, and the function of the MGR classification system is to divide music into different types according to style. In this way, the system can recommend music for users according to their interests and hobbies, allowing conveniently users to quickly retrieve and efficiently manage their favorite music. National instrumental music generally integrates many national music genres. Different national instrumental music represents various musical national schools (Tapson et al., 2018). Therefore, with the traditional cognition of Chinese instrumental music as an example, the national music schools are studied. MGR includes music signal pre-processing, feature extraction, and recognition. The goal of pre-processing is to promote the next feature extraction. Music signals contain a lot of redundant data, and the extracted characteristic parameters are the expression of music information in other ways. If the time-domain audio signal directly enters the music identification system, the amount of data will be destructive. Then, the extracted parameters are put into the classifier. After adjusting the classifier parameters, the feature model is established, and the best model formed is used to identify the type of test music samples. Therefore, the DBN is used to further study the basic characteristics of each music type. Then, softmax regression is used to predict and detect the music type of music samples (Wang et al., 2018).

(1) Software and hardware test environment

MATLAB (Matrix &Laboratory) programming software 2016A (Gevorkyan et al., 2019) is used to extract the characteristics of the original music signal. Based on the Python language, the DBN constructed by the Theano library (Budhi et al., 2021) is used, and Spyder (Baydogan and Aatas, 2021) is used as the programming environment. The identification and classification of folk musical instruments were tested on the Windows 7 64-bit operating system of the workstation Dell Precision Tower7910 (Sun et al., 2021). The main workstation parameters are as follows: Intel (R) Xeno (R) CPU E5-2620 v3 @ 2.40 GHz (8 cores) processor, 64 GB memory, NVIDIA GeForce GTX TITAN X graphics card, 1 T disk capacity.

(2) National musical instruments library

The Chinese traditional music used in the experiment is pure music from the Internet. There are five national musical instruments: erhu, guzheng, pipa, hulusi, and flute. The coding format and sampling frequency of digital music have a great impact on data analysis and processing (Youngblood, 2019). In the experiment, the music signal of traditional musical instruments was converted into WAV mono, and the sampling frequency of 22.05 kHz was used. The sampling frequency of music data from online and offline national instrumental music is 44.1 KHz, so downsampling is required (Wang et al., 2022). Due to the use of a 22.05 kHz sampling frequency, the efficiency of the algorithm can be improved without losing the basic characteristics of instrumental music. In addition, because the music download tool for each clip contains many silent clips and pure music clips, it is possible to eliminate some of the silence and assemble the pure music clips while maintaining the same sampling rate and coding style. Then, the music material is cut into 30-s segments. CoolEdit (Abbasi et al., 2020) is used to process the music materials of the original musical instrument.

There are five kinds of national musical instruments used in the experiment: erhu, guzheng, pipa, loofah, and flute. Each instrument music type includes 100 30-s wav format 500 music clips. In the music, there are 60 pieces of music for each instrument used for training: 20 for verification and 20 for testing.

(3) Music signal pre-processing of national musical instruments

The label of musical instruments is of great significance to the classification of music types and can be used to predict the emotions and music scenes contained in the music. Therefore, the recognition and classification of musical instruments also occupy an important position in the field of music information retrieval. If people are familiar with the musical instrument used to perform a particular kind of music, the effect of automatic music recognition and classification can be optimized according to the characteristics of the musical instrument used. The pre-processing process of each type of music includes pre-processing, dividing, and adding windows (Cai and Zhang, 2022). A processing method called pre-weighting has been proposed to improve the high-frequency resolution of music signals and analyze the spectrum of the whole frequency band. A first-order digital filter usually performs pre-weighting. The transfer function of the filter is expressed as


(5) $$H\,z=1-\alpha \,z^{-1}$$

The pre-emphasis factor α is generally decimal, close to 1. Let the sampling value of the genre music signal at time *n* be *x* (*n*), then the pre-emphasis data is


(6) y⁢(n)=x⁢(n)-α⁢x⁢(n-1)

**FIGURE 2:**
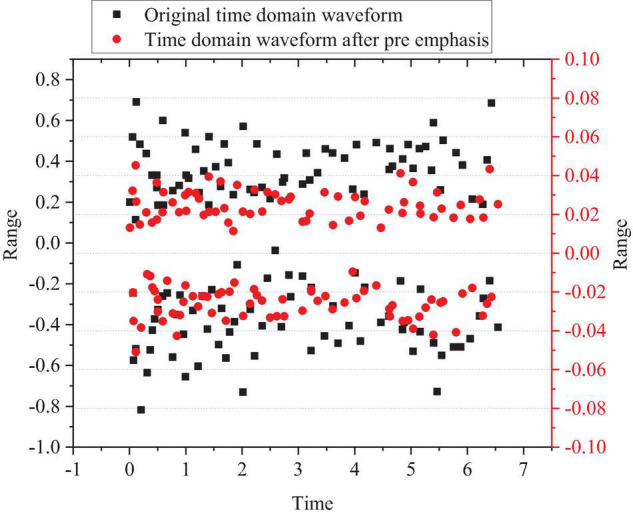
Comparison between time-domain waveform after pre-emphasis of pipa music segment and the original time-domain waveform.

Taking a music segment played with the pipa as an example, the comparison between the pre-emphasized time domain waveform of the segment and the original time-domain waveform is shown in Figure 2.

While the music genre signal is unstable, it can be regarded as static for a brief period of time. The music signal feature extraction here is based on the steady-state signal. Therefore, before the music signal feature extraction, it is generally necessary to divide the frame (Qin et al., 2021). In order to ensure that the information between the two signals during a seamless transition is not lost, it is necessary to ensure that the two signals have overlapping parts. The length of the overlap is called the frameshift. Theoretically, the calculation equation of the number of image signals of music segments is as follows:


(7) $$N=\left[\frac{N_{1}-N_{0}}{N_{2}-N_{0}}\right]$$

**FIGURE 3:**
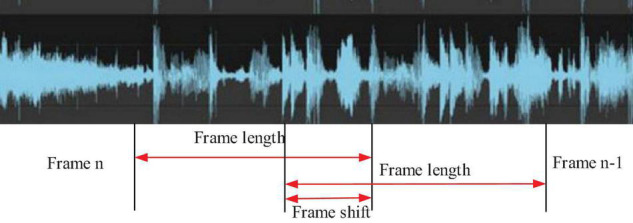
Framing diagram of the pipa music clip.

The number of frames is represented by *N*, the frameshift is represented by *N_0_*, the total signal length is represented by *N_1_*, and the frame length is represented by *N_2_*. In the test, the frame length is set to 2 s, and the frameshift is set to one-third of the frame length. A piece of music played with pipa as an example is shown in Figure 3, which is a frame diagram.

When all types of music clips are framed, in order to reduce the edge effect, increase the continuity between frames, and reduce the leakage spectrum, the music signal after frame separation also needs window processing (Kadiri and Alku, 2019). The window functions often used in audio signal processing include rectangular windows, Hanning windows, and Hamming windows.

Rectangular window:


(8) $$w\,\left(n\right)=\left\{ \begin{array}{cc} 1 & 0\leq n\leq M-1\\ 0 & o\,t\,h\,e\,r \end{array}\right.$$

Hanning window:


(9) $$w\,\left(n\right)=\left\{ \begin{array}{cc} 0.5\,\left(1-\cos\left(2\,\pi \,n/\left(M-1\right)\right)\right) & 0\leq n\leq M-1\\ 0 & o\,t\,h\,e\,r \end{array}\right.$$

Hamming window:


(10) $$w\,\left(n\right)=\left\{ \begin{array}{cc} 0.54-0.46\,\cos\,\left(2\,\pi \,n/\left(M-1\right)\right) & 0\leq n\leq M-1\\ 0 & o\,t\,h\,e\,r \end{array}\right.$$

The window length *M* of the window function has a great impact on the signal. When *M* is small, the change in short-term signal energy is obvious, making it impossible to create relatively stable short-term information signal. When *M* is large, the window function is similar to a very narrow low-pass filter, with essentially few changes in the short-term signal information, leaving it almost unchanged. Therefore, the window length cannot be too small when selecting the window function.

(4) Extracting the original characteristic parameters of Chinese traditional instrumental music

**FIGURE 4:**
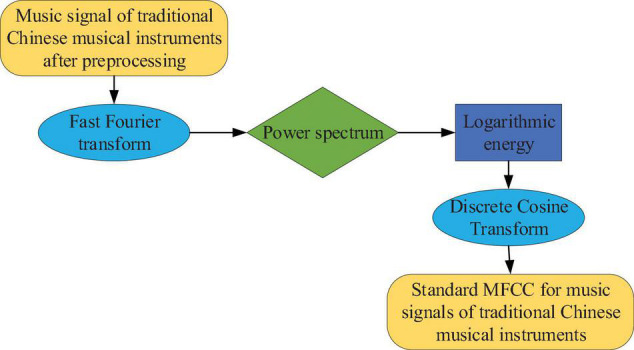
The standard MFCC parameter extraction process for traditional instrumental music.

Musical features can be used to characterize the essential attributes of redundant music. Therefore, extracting music features is an extremely important link in recognizing and classifying music genres and traditional Chinese musical instruments. The extracted original feature is the mel frequency cepstral coefficient (MFCC) (Yang et al., 2020). Since the tones of musical instruments are used to distinguish different types of musical instruments, MFCC has proven to be a representation of musical tones (Abeysinghe et al., 2021). Figure 4 shows the process of extracting characteristic parameters from traditional instrumental music MFCC.

When extracting the MFCC standard coefficient, the dimension of the MFCC standard coefficient is determined by the logarithmic energy discrete cosine transform (Sawant and Manoharan, 2020). The standard MFCC only reflects the static characteristics of musical instrument music signals. Only by combining static and dynamic features can the classification effect be more effective and comprehensive. Therefore, in addition to the standard MFCC, the experiment also extracts the second- and first-order difference parameters.

**FIGURE 5:**
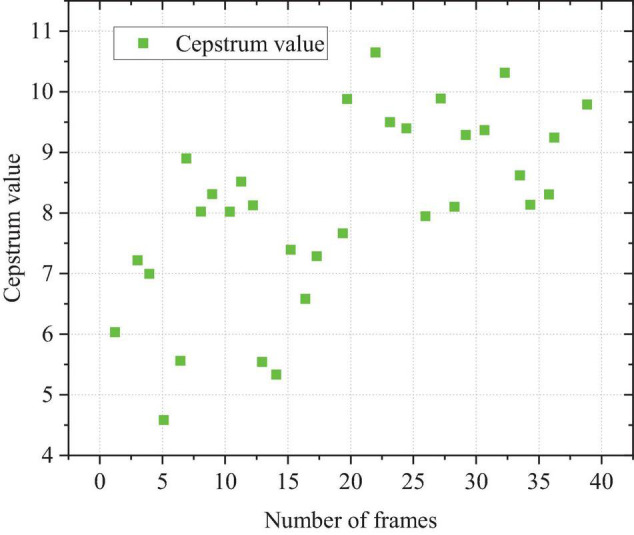
The first-dimensional MFCC characteristic parameters of the guzheng music segment.

Because the filter used in the test is divided into 12 triangles and the cosine discrete coefficient transformation is 12, the standard coefficient has 13 dimensions. With the guzheng music clip as an example, the characteristic MFCC parameters of the 30 s in the first dimension are shown in Figure 5.

The curve of parameter characteristics is shown in Figure 5. Different frames have the same dimension, and the same guzheng music segment has a MFCC characteristic parameter curve with a large peak change, indicating that there is a significant difference between different frames of guzheng music, and the attribute of this feature can be used to describe the music played by the instrument (Wahyuni et al., 2021). Since the size of the curve of MFCC parameter features is different, all 39-dimensional vector features are used to reflect the characteristics of guzheng music fragments.

(5) The DBN-based national musical instrument recognition algorithm

**FIGURE 6:**
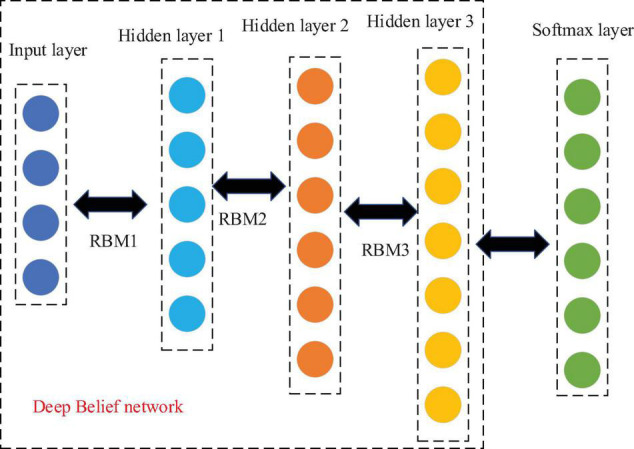
The DBN-based structure of the national musical instrument recognition and differentiation network.

In this study, the music genre discrimination algorithm based on the DBN was experimentally studied. For the distinguishing feature vector of national musical instruments, the vector feature of the MFCC network is selected as the input. The sample is realized by a 2-s long musical instrument signal, which is then input into the three hidden layers of the DBN. Then, the software output layer outputs the label prediction of the musical instrument. Figure 6 shows the basic structure of the network:

The feature input dimension of the network is 39 dimensions; therefore, each layer has fewer nodes. It is not necessary to use the dropout method (Chen et al., 2020) to prevent overshoot, but the strategy to prevent overshoot is early stopping (Li et al., 2022). Early stopping refers to the checking of the changes of model performance evaluation indicators at the end of each epoch traversed during fine-tuning the network (an epoch refers to traversing all training data, including training set and verification set once). If the performance of the model is no longer improving, it is essential to stop training. The main problem is how to determine that the model’s performance has not improved. In this experiment, the accuracy of the validation set was used as the evaluation index. However, the failure to improve the accuracy rate does not imply that it will not increase again whenever the accuracy rate decreases. The reason for this is that accuracy may decrease after the current epoch but will improve after the next epoch training. Therefore, it is impossible to determine whether or not the accuracy is improved by decreasing one time or two times in a row, but only to record the best accuracy of the verification set thus far in the training process. If the best accuracy is not achieved after 10 or more consecutive drops, the accuracy has not improved and the iteration will terminate.

This method is used to avoid excessive adjustments, and 100 times is set as the upper limit. However, in fact, the verification set reached the highest level of accuracy after 89 epochs; hence, 89 is the ideal epoch number. After determining the number of iterations, the number of small-batch processing blocks and the learning rate are determined, different hidden layers and nodes are selected, and their effects on the discrimination accuracy and the prediction accuracy of national instruments are compared.

### The Deep Belief Network-Based Music Genre Learning Platform

**FIGURE 7:**
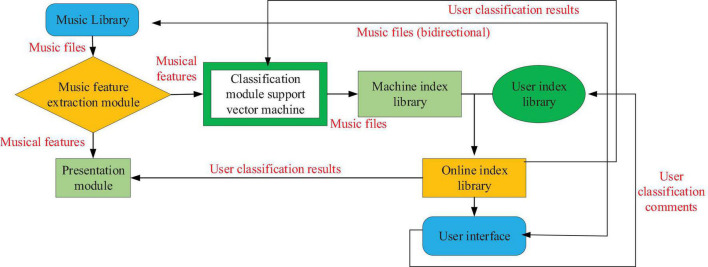
The retrieval framework of differentiated music library platform.

The platform has two main functions: distinguishing music collections by genre and presenting similarities by genre to facilitate research. These two functions are based on the unique characteristics of the music genre and use supervised learning techniques. The system can understand the user’s views on music ratings so that their own ratings and performance results are in line with the user’s preferences. The architecture of distinguishing music library platform search is shown in Figure 7:

When the system starts, a set of training data, including music files and their corresponding types, is required to form a presentation module and an initial differentiation module. After the initial training of the module, all processed music features are input into the display and differentiation module. The discrimination results of the discrimination module (i.e., corresponding music files and corresponding music genres) are recorded in the index library of the machine and copied to the online index library for user retrieval. The results of the rendering module mapping can be transmitted to the user interface. At this stage, the system can be opened to users. Users can find and listen to music and other text information, enter composers, names, singers, or music genres to find or browse the whole music library, and select their favorite music through the music content displayed on the platform.

## Results and Discussion

### Prediction Accuracy of the Deep Belief Network With Different Hidden Layers

**FIGURE 8:**
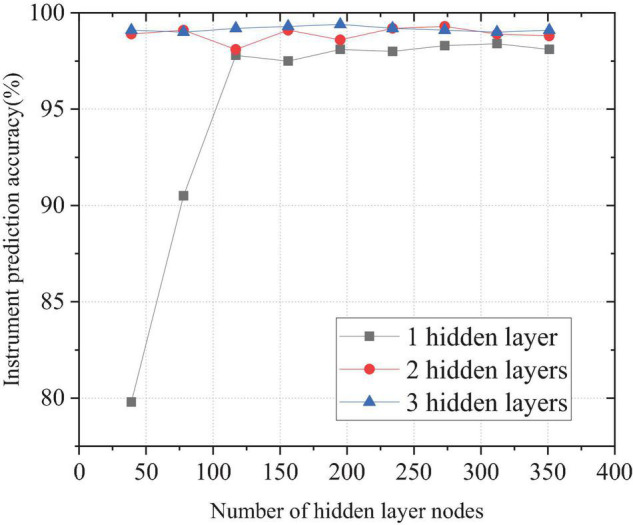
Comparison of prediction accuracy of different hidden layers for national musical instruments.

When the DBN contains one, two, and three hidden layers, the number of neuron nodes changes. Figure 8 shows the prediction accuracy of national instrumental music.

Figure 8 shows that, when the DBN contains only one hidden layer node number, the accuracy is basically the same as when the number of neural nodes in the hidden layer is 117. Then, with the increase in the number of neural nodes, the accuracy is maintained at approximately 98%. When there are two hidden layers, the number of nodes in the first hidden layer 1 is 117. The change in the number of neurons in the second layer has little effect on the accuracy, and the accuracy fluctuates approximately 99%. When the DBN has three hidden layers, the number of nodes in the first layer is 117, and the number of nodes in the second layer is 78. The change in the number of neurons in the third layer has little effect on the prediction accuracy, and the prediction accuracy is 99.1%. The comparison of the results in Figure 8 illustrates that the first layer of the hidden layer has a greater impact on the prediction results. Network performance convergence basically occurs when the number of nodes hidden in the first layer is approximately three times the characteristic dimension of the sample. The number of neural nodes in other hidden layers has little effect on national instrumental music discrimination accuracy, but the performance improves slightly with the increase of layers. Meanwhile, with the increase in the number of nerve nodes in each layer, the training time will also increase. Therefore, the number of nerve nodes in other hidden layers does not need to be too much except in the first layer.

According to the above research, the optimal parameters of the network model used are as follows. The number and size of mini-batch blocks during training is 50. The number of input layer nodes is 39. As the Softmax layer outputs five types of national musical instruments, the number of nodes in the output layer is five. During the pre-training of RBM, Gibbs sampling updates the parameters every time; the step size is 1, the number of iterations is 10, and the learning speed update is 0.001. The number of iterations of the back-propagation algorithm is 100, and the update rate of the learning weight is 0.1.

### Performance Comparison of Ethnic Music Genre Recognition Based on Traditional Methods and the Deep Belief Network

**FIGURE 9:**
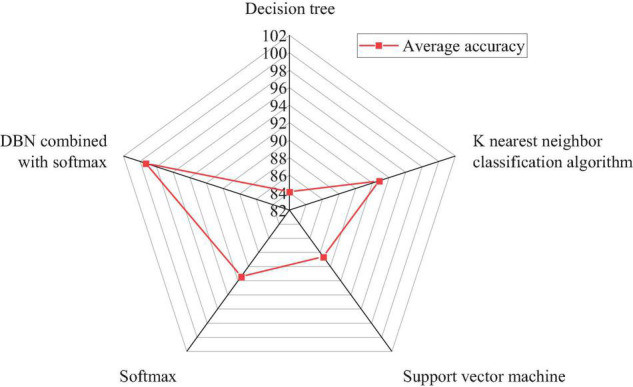
The average accuracy of the discrimination algorithm based on the DBN and traditional discriminator for national musical instrument recognition.

Each kind of instrumental music includes 2,160 samples, with a total of 10,800 samples. Each instrument has 670 samples, with a total of 3,350 instrument samples. The first step is to mark all instrument samples and then use the training model to cross-check the verification set and predict the instrument samples for the test set. Finally, the actual instrument tag is compared with the predicted instrument tag to obtain the average recognition rate. The DBN-based discrimination algorithm, the KNN discrimination algorithm (Abu Alfeilat et al., 2019), a traditional decision tree discriminator, the softmax function, and support vector machine (SVM) are used to train, test, and detect five national musical instruments, respectively. Figure 9 depicts the average accuracy of recognition and discrimination.

Figure 9 reveals that inputting MFCC features directly into the traditional discriminator results in poor recognition and discrimination. The decision tree discriminator has the lowest accuracy of 84.1%, while KNN has the highest accuracy of 92.8%. The abstract features are obtained through the further study of the DBN. The softmax layer has the best distinguishing effect on national musical instruments, with an accuracy of 99.3%, and KNN is 6.5% lower. The experimental results show that, through the further study of DBN, the Chinese traditional music samples can obtain better recognition and discrimination effect.

### Confusion Matrix Based on General Classification Method and the Deep Belief Network for Identification and Distinction of National Musical Instruments

**FIGURE 10:**
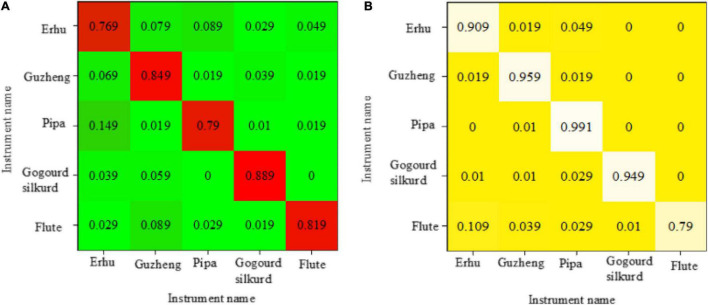
The confusion matrix composed of recognition rates [**(A)** recognition rate confusion matrix of distinguishing national musical instruments by distinguishing trees; **(B)** recognition rate confusion matrix of distinguishing national musical instruments by KNN].

Figure 10A depicts the confusion matrix composed of the recognition rates of five national musical instruments, which is obtained by identifying and distinguishing them using different methods. The abscissa axis represents the actual name of the instrument, whereas the ordinate axis represents the expected name of the instrument. The number in the matrix shows the actual proportion of samples of real labels identified as predicted instruments. The probability of correct recognition is represented by diagonal numbers, and the recognition accuracy gradually increases with the color becoming darker. Figure 10B shows a confusion matrix of recognition accuracy obtained by directly inputting the 39-dimensional MFCC feature of each instrument sample into KNN.

Figure 10A is the recognition accuracy confusion matrix obtained by directly capturing the 39-dimensional MFCC features of each traditional Chinese instrumental sample in the decision tree. It reveals that the discrimination accuracy of identifying national musical instruments according to this method is greater than 80%, where the recognition accuracy of the erhu is very weak (76.9%) and that of the flute is the highest (88.9%). Figure 10B shows that the pipa has a recognition accuracy of 99.1%, the remaining musical instruments have a recognition accuracy of more than 90%, and the flute has a recognition accuracy of 79%.

**FIGURE 11:**
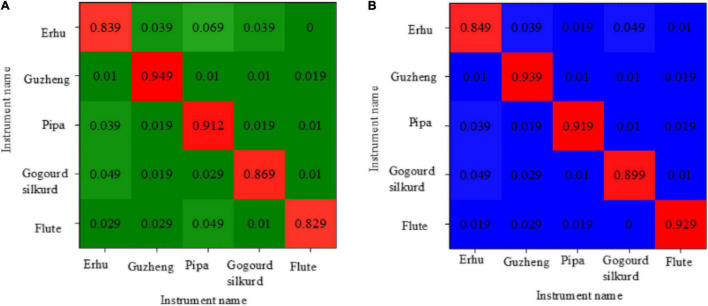
The confusion matrix of recognition accuracy [**(A)** the recognition rate confusion matrix of SVM for distinguishing the national musical instruments; **(B)** the recognition rate confusion matrix of softmax for distinguishing the national musical instruments].

Figure 11A shows the recognition accuracy confusion matrix obtained by directly capturing the 39-dimensional MFCC features of each instrument sample in SVM. Figure 11B depicts the recognition accuracy confusion matrix obtained by directly capturing the 39-dimensional MFCC features of each instrument sample in the softmax neural network.

Figure 11A shows that, when this method is used to distinguish national musical instruments, the recognition rate of the guzheng is 94.9%, whereas the recognition rate of the flute and the erhu is low, which are less than 85%. Figure 11B shows that the recognition accuracy of the erhu is lower than 84.9%, and the highest recognition accuracy of the guzheng is 93.9%.

**FIGURE 12:**
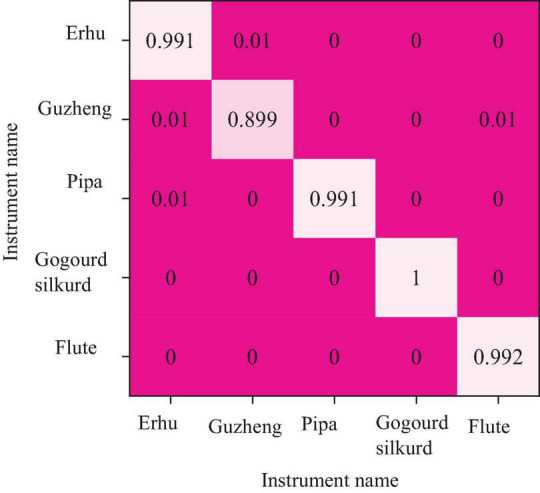
The confusion matrix of recognition accuracy of the DBN and softmax in distinguishing the national musical instruments.

Figure 12 shows the recognition accuracy confusion matrix obtained by combining the MFCC features of 39 sizes of each instrument sample in the DBN with neural network softmax. This method has the best recognition and discrimination effect on national musical instruments, and the average correct recognition and discrimination rate exceeds 99%. The recognition accuracy of the guzheng is the lowest at 89.9%, while all flutes have a 100% recognition rate.

The recognition accuracy of five discrimination methods from the confusion matrix is compared. The recognition effect of various methods to distinguish various national musical instruments is pointed out. This may be related to the internal characteristics of the discriminator formation of these musical instruments. The erhu and the flute do not have high recognition accuracy in traditional distinction and recognition. However, after the DBN retrained the instruments on the sample, the recognition rate of these two instruments also increased significantly, which proves the superiority of the DBN in the identification and differentiation of national instruments. The most basic musical characteristics of national instrumental music can be extracted using this method.

## Conclusion

With the rapid development of computers, people’s research on neural networks is increasingly transitioning from shallow structures to deep structures. In particular, DL has become a hot research topic in recent years. With the proliferation of multimedia audio data, how to retrieve the required music quickly and accurately has become an urgent question in the field of music information search. Therefore, the DL and music information search research is quite meaningful and useful. This exploration introduces the relevant theories in the field of music recognition and discrimination, including classical discrimination methods, relevant DL knowledge, and manual feature extraction. The MGR classification studied, and the unique recognition and classification of Chinese traditional musical instruments are two important parts in the field of music information retrieval. This exploration attempts to combine it with DL knowledge, apply the DL network model to the feature extraction task of music genres and Chinese traditional musical instruments, and then realize the recognition and classification of music. The experimental results show that the algorithm is more accurate in traditional national musical instrument recognition, proving the platform’s superiority. However, there are still some research deficiencies. For example, the instrument library used in the experiment is slightly simpler and only contains monotonous instruments. Future studies might attempt to distinguish the categories of musical instruments from the music played by various musical instruments using DL. In addition, the experiments used the DBN to create the recognition and classification system. The difference is in the internal structure of the network, such as the number of network layers, the number of neuron nodes, and network optimization strategies. Besides, the setting of parameters during training also differs. Other DL algorithms can be adopted in the future to solve these two tasks, such as the convolutional neural network, convolutional DBN, and other single or composite deep neural networks.

## Data Availability Statement

The raw data supporting the conclusions of this article will be made available by the authors, without undue reservation.

## Ethics Statement

The studies involving human participants were reviewed and approved by the Henan Normal University Ethics Committee. The patients/participants provided their written informed consent to participate in this study. Written informed consent was obtained from the individual(s) for the publication of any potentially identifiable images or data included in this article.

## Author Contributions

The author confirms being the sole contributor of this work and has approved it for publication.

## Conflict of Interest

The author declares that the research was conducted in the absence of any commercial or financial relationships that could be construed as a potential conflict of interest.

## Publisher’s Note

All claims expressed in this article are solely those of the authors and do not necessarily represent those of their affiliated organizations, or those of the publisher, the editors and the reviewers. Any product that may be evaluated in this article, or claim that may be made by its manufacturer, is not guaranteed or endorsed by the publisher.
